# Supplementary material for: Molecular Characterization of Associated Pathogens in Febrile Patients during Inter-Epidemic Periods of Urban Arboviral Diseases in Tapachula Southern Mexico
Source: Pathogens. 2021 Nov 8;10(11):1450. doi: 10.3390/pathogens10111450 (PMC8618676; doi:10.3390/pathogens10111450)
Supplement: Supplementary file 1 [file pathogens-10-01450-s001.zip › pathogens-1434546-supplementary.pdf]

Table S1. Demographic data of the 253 patients included in this study.

Population Data	N (%)	DENV (%)	CHIK (%)	ZIKA (%)	CHIK/ZIKA (%)	Leptospira (%)	Total Positives (%)
April–June 2015							
**Age Group**							
0–20	19 (7.51)	1 (0.40)	16 (6.32)	0 (0)	0 (0)	0 (0)	17 (6.71)
21–30	30 (11.86)	0 (0)	27 (10.67)	1 (0.40)	0 (0)	0 (0)	28 (11.07)
31–40	45 (17.79)	0 (0)	40 (15.81)	5 (1.98)	0 (0)	0 (0)	45 (17.79)
41–50	64 (25.30)	0 (0)	58 (22.92)	4 (1.58)	0 (0)	2 (0.79)	64 (25.30)
>50	48 (18.97)	0 (0)	42 (16.60)	2 (0.79)	0 (0)	1 (0.40)	45 (17.79)
Subtotal	206 (81.42)	1 (0.40)	184 (72.73	12 (4.74)	0 (0)	3 (1.19)	200 (79.05)
**Gender**							
Female	138 (54.55)	1 (0.40)	125 (49.41)	7 (2.77)	0 (0)	3 (1.19)	136 (53.75)
Male	68 (26.88)	0 (0)	58 (22.92)	5 (1.98)	0 (0)	0 (0)	63 (24.90)
February–March 2016							
**Age Group**							
0–20	7 (2.77)	0 (0)	2 (0.79)	3 (1.19)	0 (0)	2 (0.79)	7 (2.77)
21–30	13 (5.14	0 (0)	2 (0.79)	5 (1.98)	1 (0.40)	2 (0.79)	10 (3.95)
31–40	11 (4.35)	0 (0)	2 (0.79)	4 (1.58)	1 (0.40)	0 (0)	7 (2.77)
41–50	8 (3.16)	0 (0)	0 (0)	4 (1.58)	2 (0.79)	0 (0)	6 (2.37)
>50	8 (3.16)	0 (0)	0 (0)	6 (2.37)	1 (0.40)	1 (0.40)	8 (3.16)
Subtotal	47 (18.58)	0 (0)	6 (2.37)	22 (8.69)	5 (1.98)	5 (1.98)	38 (15.01)
**Gender**							
Female	31 (12.25)	0 (0)	5 (1.98)	13 (5.13)	3 (1.19)	3 (1.19)	24 (9.48)
Male	16 (6.32)	0 (0)	1 (0.40)	9 (3.56)	2 (0.79)	2 (0.79)	14 (5.53)
Total Women, 169 (66.8); Total Mens, 84 (33.2); Global Total, 253 (100).							

Table S2. Demographic information of pathogen localities in febrile patients.

		Samples Collected, No.				
**Collection Site**	**Samples Collected, No.**	**Positive for CHIKV**	**Positive for ZIKV**	**co-Infection Positive for CHIKV/ZIKV**	**Positive for Leptospira**	**Positive for DENV**
Acacoyagua	1	1	–	–	–	–
Cacahoatan	23	18	4	–	–	–
Huehuetan	24	20	3	–	1	–
Huixtla	23	20	1	–	–	–
Mazatan	2	2	0	–	–	–
Metapa de Dominguez	1	1	0	–	–	–
Suchiate	4	4	0	–	–	–
Tapachula	132	91	22	3	6	1
Tuxtla Chico	39	30	3	2	1	–
Tuzantan	2	1	1	–	–	–
Villa Comaltitlan	2	1	0	–	–	–
**Gender**						
Female	169	130	20	3	6	1
Male	84	59	14	2	2	
**Total**	253	189	34	5	8	1

Table S3. Comparison of clinical variables between the CHIKV and ZIKV groups.

SYMPTOM	ZIKV+ / n=35 (%)	CHIKV+ / n=189 (%)	[a] *p-Value*
Headache	33 (94.3)	173 (91.5)	0.746
Myalgia *	23 (65.7)	22 (11.6)	<0.01
Mild-Moderate Arthralgia *	25 (71.4)	76 (40.2)	<0.01
Severe Polyarthralgia *	10 (28.6)	112 (59.3)	<0.01
Arthritis *	6 (17.1)	103 (54.5)	<0.01
Retroocular Pain *	18 (51.4)	30 (15.9)	<0.01
Exanthema *	21 (60.0)	151 (79.9)	<0.01
Pruritus	22 (62.9)	138 (73.0)	0.227
Vomit	3 (8.6)	27 (14.3)	0.588
Sickness	23 (65.7)	116 (61.4)	0.707
Shivers	32 (91.4)	175 (92.6)	0.734
Photophobia	1 (2.9)	2 (1.1)	0.401
Abdominal Pain	11 (31.4)	81 (42.9)	0.262
Diarrhea	9 (25.7)	73 (38.6)	0.182
Conjunctivitis *	25 (71.4)	20 (10.6)	<0.01
Nasal Congestion	8 (22.9)	24 (12.7)	0.12
Cough *	15 (42.9)	34 (18.0)	<0.01
Pharyngitis	11 (31.4)	70 (37.0)	0.571
Taste Aleration *	15 (42.9)	150 (79.4)	<0.01
Adenomegaly *	9 (25.7)	85 (45.0)	<0.05
Inflammation Of The Eyelid	3 (8.6)	8 (4.2)	0.384

Dyspnoea	3 (8.6)	6 (3.2)	0.151
Cardiac Alteration	0 (0)	2 (1.1)	1
Disorientation	0 (0)	1 (0.5)	1
Muscular Weakness	4 (11.4)	30 (15.9)	0.615
Otitis	0 (0)	1 (0.5)	1
Petequias	1 (2.9)	2 (1.1)	0.401
Edema *	5 (14.3)	70 (37.0)	<0.05

Lanciotti et al (2008)	Primer sentido (ZIKV 1086)	Sonda (ZIKV 1107)	Primer anti-sentido (ZIKV 1162c)
	CCGCTGCCCAACACAAG	AGCCTACCTTGACAAGCAGTCAGACACTCAA	ATGTCTGCAAAAGAACGTTAGTGG
BrazilAmniotic2015	CCGCTGCCCAACACAAG	AGCCTACCTTGACAAGCAATCAGACACTCAA	ATGTCTGCAAAAGAACGTTAGTGG
BrazilBrain2015	CCGCTGCCCAACACAAG	AGCCTACCTTGACAAGCAATCAGACACTCAA	ATGTCTGCAAAAGAACGTTAGTGG
Brazil2015_1	CCGCTGCCCAACACAAG	AGCCTACCTTGACAAGCAATCAGACACTCAA	ATGTCTGCAAAAGAACGTTAGTGG
Brazil2015_2	CCGCTGCCCAACACAAG	AGCCTACCTTGACAAGCAATCAGACACTCAA	ATGTCTGCAAAAGAACGTTAGTGG
Brazil2015_3	CCGCTGCCCAACACAAG	AGCCTACCTTGACAAGCAATCAGACACTCAA	ATGTCTGCAAAAGAACGTTAGTGG
Brazil2015_4	CCGCTGCCCAACACAAG	AGCCTACCTTGACAAGCAATCAGACACTCAA	ATGTCTGCAAAAGAACGTTAGTGG
Brazil2015_5	CCGCTGCCCAACACAAG	AGCCTACCTTGACAAGCAATCAGACACTCAA	ATGTCTGCAAAAGAACGTTAGTGG
Cambodia2010	CCGCTGCCCAACACAAG	AGCCTACCTTGACAAGCAATCAGACACTCAA	ATGTCTGCAAAAGAACGTTAGTGG
CAR1968	TCGCTGCCCAACACAAG	AGCCTACCTTGACAAGCAATCAGACACTCAA	ATGTTTGCAAAAGAACATTGGTGG
CAR1976	TCGCTGCCCAACACAAG	AGCCTACCTTGATAAGCAATCAGACACCCAA	ATGTGTGCAAAAGAACATTGGTGG
CAR1979	TCGCTGCCCAACACAAG	AGCCTACCTTGATAAGCAATCAGACACCCAA	ATGTGTGCAAAAGAACATTGGTGG
CAR1980	TCGCTGCCCAACACAAG	AGCCTACCTTGACAAGCAATCAGACACTCAA	ATGTATGCAAAAGAACATTGGTGG
FrenchPolynesia2013	CCGCTGCCCAACACAAG	AGCCTACCTTGACAAGCAATCAGACACTCAA	ATGTCTGCAAAAGAACGTTAGTGG
Guatemala2015_1	CCGCTGCCCAACACAAG	AGCCTACCTTGACAAGCAATCAGACACTCAA	ATGTCTGCAAAAGAACGTTAGTGG
Guatemala2015_2	CCGCTGCCCAACACAAG	AGCCTACCTTGACAAGCAATCAGACACTCAA	ATGTCTGCAAAAGAACGTTAGTGG
Haiti2014	CCGCTGCCCAACACAAG	AGCCTACCTTGACAAGCAATCAGACACTCAA	ATGTCTGCAAAAGAACGTTAGTGG
Malaysia1966	CCGCTGCCCAACACAAG	AGCCTACCTTGACAAGCAGTCAGACACTCAA	ATGTTTGCAAAAGAACGTTAGTGG
Martinique2015	CCGCTGCCCAACACAAG	AGCCTACCTTGACAAGCAATCAGACACTCAA	ATGTCTGCAAAAGAACGTTAGTGG
Nigeria1968	TCGCTGCCCAACACAAG	AGCCTACCTTGACAAGCAGTCAGACACTCAA	ATGTCTGTAAAAGAACATTGGTGG
Philippines2012	CCGCTGCCCAACACAAG	GGCCTACCTTGACAAGCAGTCAGACACTCAA	ATGTCTGCAAAAGAACGTTAGTGG
PuertoRico2015	CCGCTGCCCAACACAAG	AGCCTACCTTGACAAGCAATCAGACACTCAA	ATGTCTGCAAAAGAACGTTAGTGG
SaoPaulo2015	CCGCTGCCCAACACAAG	AGCCTACCTTGACAAGCAATCAGACACTCAA	ATGTCTGCAAAAGAACGTTAGTGG
Senegal1968	CCGCTGTCCAACACAAG	AGCCTACCTTGACAAGCAGTCAGACACTCAA	ATGTCTGCAAGAGAACATTGGTGG
Senegal1984	CCGTTGTCCAACACAAG	AGCCTACCTTGACAAGCAGTCAGACACTCAA	ATGTCTGCAAGAGAACATTGGTGG
Senegal1997	CCGCTGTCCAACACAAG	AGCCTACCTTGACAAGCAATCAGACACTCAA	ATGTCTGCAAAAGAACATTAGTGG
Senegal2001	TCGTTGCCCAACACAAG	AGCCTACCTTGACAAGCAATCAGACACTCAA	ATGTCTGCAAAAGAACATTAGTGG
Suriname2015	CCGCTGCCCAACACAAG	AGCCTACCTTGACAAGCAATCAGACACTCAA	ATGTCTGCAAAAGAACGTTAGTGG
Thailand2013	CCGCTGCCCAACACAAG	AGCCTACCTTGACAAGCAATCAGACACTCAA	ATGTCTGCAAAAGAACGTTAGTGG
Thailand2014	CCGCTGCCCAACACAAG	AGCCTACCTTGACAAGCAATCAGACACTCAA	ATGTCTGCAAAAGAACGTTAGTGG
Uganda1947_1	TCGTTGCCCAACACAAG	AGCCTACCTTGACAAGCAATCAGACACTCAA	ATGTCTGCAAAAGAACATTAGTGG
Uganda1947_2	TCGTTGCCCAACACAAG	AGCCTACCTTGACAAGCAATCAGACACTCAA	ATGTCTGCAAAAGAACATTAGTGG
Yap2007	CCGCTGCCCAACACAAG	AGCCTACCTTGACAAGCAGTCAGACACTCAA	ATGTCTGCAAAAGAACGTTAGTGG
Mexico2016_1	CCGCTGCCCAACACAAG	AGCCTACCTTGACAAGCAATCAGACACTCAA	ATGTCTGCAAAAGAACGTTAGTGG
Mexico2016_2	CCGCTGCCCAACACAAG	AGCCTACCTTGACAAGCAATCAGACACTCAA	ATGTCTGCAAAAGAACGTTAGTGG
Mexico2016_3	CCGCTGCCCAACACAAG	AGCCTACCTTGACAAGCAATCAGACACTCAA	ATGTCTGCAAAAGAACGTTAGTGG
Mexico2016_4	CCGCTGCCCAACACAAG	AGCCTACCTTGACAAGCAATCAGACACTCAA	ACGTCTGCAAAAGAACGTTAGTGG
Mexico2016_5		AGCCTACCTTGACAAGCAATCAGACACTCAA	ATGTCTGCAAAAGAACGTTAGTGG
Mexico2015_2	CCGCTGCCCAACACAAG	AGCCTACCTTGACAAGCAATCAGACACTCAA	ATGTCTGCAAAAGAACGTTAGTGG

Figure S1. **Alignment primers/probes for ZIKV**. All primers/probes are in the 5′ to 3′ direction. Mismatches are highlighted with blue boxes. The antisense primer sequence is inversely complemented. This primer/probe set targets the envelope gene that is capable of detecting all isolates from Asia, South America, and Mexico. However, there are 1–7 mismatches that can alter the efficiency of the assay.

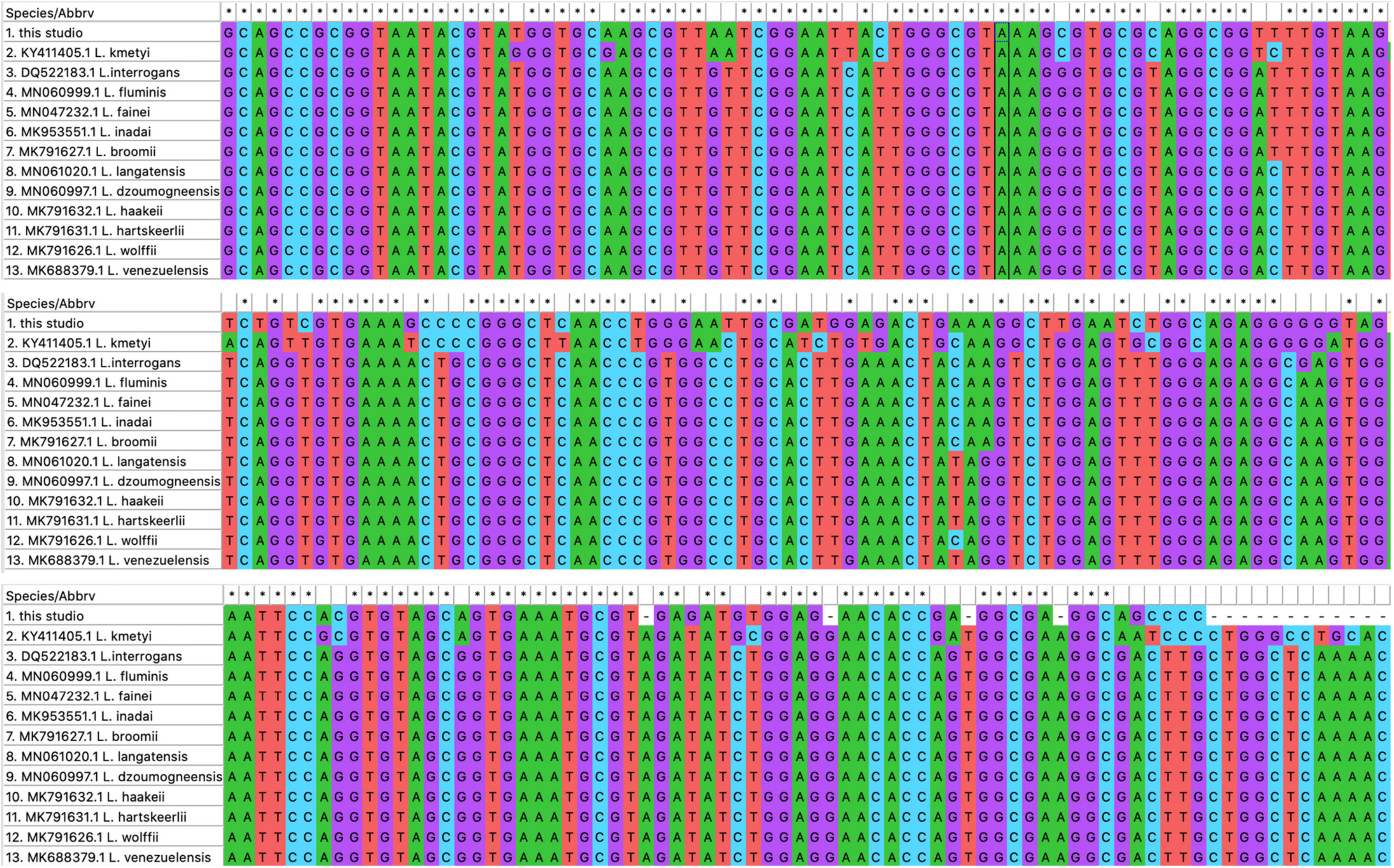
Species/Abbrv
1. this studio
2. KY411405.1 L. kmetyi
3. DQ522183.1 L.interrogans
4. MN060999.1 L. fluminis
5. MN047232.1 L. fainei
6. MK953551.1 L. inadai
7. MK791627.1 L. broomii
8. MN061020.1 L. langatensis
9. MN060997.1 L. dzoumogneensis
10. MK791632.1 L. haakeii
11. MK791631.1 L. hartskeerlii
12. MK791626.1 L. wolffii
13. MK688379.1 L. venezuelensis

Figure S2. **Multiple alignment of leptospira species.** Multiple alignment of partial sequence of the 16S ribosomal RNA gene of leptospira species reported in GenBank. Identical bases are shaded the same color, asterisks show 100% identity in the diferent sequences. The BLAST match showes 87.38% identity with L. Kmetyi (KY411405.1) and ~ 80% for the other leptospira species.
